# Effects of Treatment Setting on Outcomes of Flexibly-Dosed Intensive Cognitive Behavioral Therapy for Pediatric OCD: A Randomized Controlled Pilot Trial

**DOI:** 10.3389/fpsyt.2021.669494

**Published:** 2021-05-17

**Authors:** Robert R. Selles, Zainab Naqqash, John R. Best, Diana Franco-Yamin, Serene T. Qiu, Jessica S. Ferreira, Xiaolei Deng, Dagmar Kr. Hannesdottir, Carla Oberth, Laura Belschner, Juliana Negreiros, Lara J. Farrell, S. Evelyn Stewart

**Affiliations:** ^1^British Columbia Children's Hospital Research Institute, Vancouver, BC, Canada; ^2^Department of Psychiatry, University of British Columbia, Vancouver, BC, Canada; ^3^Gerontology Research Centre, Simon Fraser University, Vancouver, BC, Canada; ^4^Department of Psychology, University of British Columbia, Vancouver, BC, Canada; ^5^Department of Psychology, Simon Fraser University, Burnaby, BC, Canada; ^6^Centre for Child Development and Behaviour, Reykjavik, Iceland; ^7^School of Applied Psychology, Griffith University, Gold Coast, QLD, Australia; ^8^Provincial Health Services Authority, British Columbia Mental Health and Substance Use Services Research Institute, Vancouver, BC, Canada

**Keywords:** stepped care, home-based treatment, exposure and response prevention, family treatment, treatment trial

## Abstract

**Introduction:** Optimizing individual outcomes of cognitive-behavioral therapy (CBT) remains a priority.

**Methods:** Youth were randomized to receive intensive CBT at a hospital clinic (*n* = 14) or within their home (*n* = 12). Youth completed 3 × 3 h sessions (Phase I) and up to four additional 3-h sessions as desired/needed (Phase II). An independent evaluator assessed youth after Phase I, Phase II (when applicable), and at 1- and 6-months post-treatment. A range of OCD-related (e.g., severity, impairment) and secondary (e.g., quality of life, comorbid symptoms) outcomes were assessed.

**Results:** Families' satisfaction with the treatment program was high. Of study completers (*n* = 22), five youth (23%) utilized no Phase II sessions and 9 (41%) utilized all four (Median Phase II sessions: 2.5). Large improvements in OCD-related outcomes and small-to-moderate benefits across secondary domains were observed. Statistically-significant differences in primary outcomes were not observed between settings; however, minor benefits for home-based treatment were observed (e.g., maintenance of gains, youth comfort with treatment).

**Discussion:** Intensive CBT is an efficacious treatment for pediatric OCD. Families opted for differing doses based on their needs. Home-based treatment, while not substantially superior to hospital care, may offer some value, particularly when desired/relevant.

**Clinical Trial Registration:** www.ClinicalTrials.gov; https://clinicaltrials.gov/ct2/show/NCT03672565, identifier: NCT03672565.

## Introduction

### Treatment of Pediatric OCD

Scientific consensus supports cognitive-behavioral therapy (CBT) utilizing exposure and response prevention (ERP) as a first line treatment for pediatric obsessive-compulsive disorder (OCD) given its safety, tolerability, and efficacy in reducing symptom severity and improving global well-being (e.g., impairment, quality of life, family functioning) (1–5). However, many challenges remain around CBT effectiveness. Approximately one third of youth do not respond to treatment and an additional proportion of youth benefit from treatment, but remain clinically impaired (4). Poor dissemination, clinician utilization, and patient access of ERP-focused CBT represent additional challenges (6–8). As a result, continued efforts to optimize CBT through novel approaches to treatment delivery are needed.

### Treatment Dose

While standardized protocols have been essential in establishing the efficacy of CBT for OCD-affected youth, fixed-dose models inadequately address inter-individual patient needs and desires, reduce efficiency of resource utilization, and thereby hold limited relevance to community care. For example, analysis of data from the NordLOTS trial found that 38% of youth were already considered responders by week 7, 73% were responders by the end of the 14-week protocol, and 50% of non-responders to the 14-week protocol responded after a second 14-week course (9, 10). Overall, a move away from standardized dose models and toward individually tailored delivery of CBT is not only warranted, but more consistent with community care models.

### Treatment Intensity

Stepped care models, in which all patients receive a low intensity treatment (e.g., bibliotherapy) and non-responders proceed to higher intensity treatments (e.g., direct CBT), have been examined as a means to optimize resource utilization; however, the benefits of this approach are limited by higher OCD-related costs (i.e., sustained impairment) associated with the delay in optimal care for individuals unlikely to respond to low-intensity interventions (11). Alternatively, leading with scalable high intensity interventions may similarly optimize resource utilization while ensuring adequate care for more severely-affected youth. Intensive CBT, in which traditional weekly CBT sessions are condensed into a shorter time frame using longer sessions and/or increased session frequency, has been associated with rapid and robust improvements, as well as similar long-term outcomes, when compared to weekly approaches (12–14). Dosing of ERP is identified as an important contributor to response (15), although time restrictions represent a primary barrier to in-session ERP utilization among clinicians (7). As such, longer session length may enhance outcomes by providing additional opportunity for ERP implementation. To date, intensive CBT has demonstrated strong potential as a brief and rapid initial intervention (14, 16) and as a cost-effective approach for treatment refractory populations (17).

### Treatment Setting

Research efforts to better understand the mechanisms through which ERP contributes to positive change in OCD-affected patients have identified the relevance of inhibitory learning (i.e., fear associations are inhibited, rather than replaced, by non-fear based associations learned during exposure) (18). Inhibitory learning appears to be impaired in OCD-affected individuals (19, 20) and the nature of these deficits appears to impact response to treatment (21). With the inhibitory learning model highlighting the importance of varying stimuli and contexts (18), providing ERP to OCD-affected youth in their natural environments (e.g., home, community), rather than in an hospital/clinic setting, may offer an opportunity to enhance outcomes. While home/community ERP was utilized with promising outcomes in Farrell et al.'s pilot trial of brief intensive CBT (16), direct comparisons of home vs. clinic CBT are lacking. A pilot trial in OCD-affected adults suggested no differences in improvement across office-vs.-home CBT delivery; however, the authors note that their clinical experiences, and discussions with providers who had incorporated or included home-based sessions into their programs, suggested that these sessions often provided unique opportunities to support patients through challenging scenarios that would not be feasible in the office and often led to meaningful change (22). Further comparison of outcomes between clinic and home ERP, particularly within a pediatric OCD population, is needed.

### Present Study

Incorporating these goals, the present study sought to implement a patient- and family-driven, flexible-dose model of intensive CBT delivery while randomizing families to receive care in home vs. hospital settings. In particular, the following aims were explored:

*Specific Aim 1*. To evaluate the efficacy of an intensive flexibly-dosed CBT program in reducing OCD-related severity, impairment, and family accommodation. Consistent with past evidence in support of intensive CBT, we hypothesized that the program would be associated with large treatment effects across primary outcomes.
*Specific Aim 2*. To examine the extent to which individuals utilized available treatment sessions across the protocol. Given past evidence of variability in response, we hypothesized that families would utilize differing proportions of available treatment sessions.
*Specific Aim 3*. To compare the efficacy, treatment utilization, and satisfaction between home and clinic settings. Given theoretical models and preliminary evidence, we hypothesized that sessions provided within the home would be associated with greater outcomes compared to sessions provided in a clinic setting.

## Materials and Methods

### Participants and Procedures

#### Study Overview

**Figure 1:**
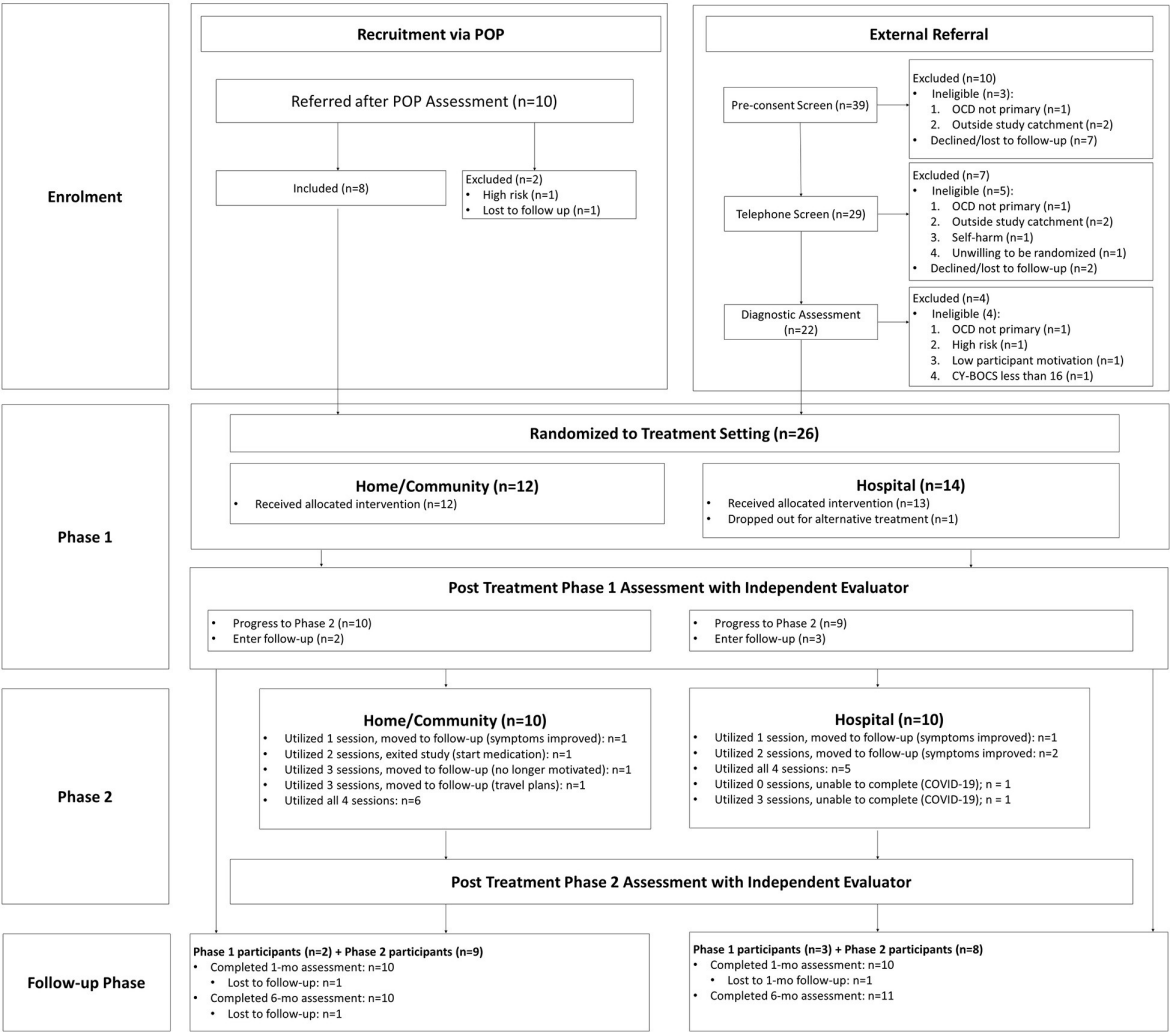
Flow chart of participants through study procedures.

The present study utilized a randomized controlled trial design to compare the utility of home/community vs. outpatient clinic setting delivery of intensive CBT for OCD-affected youth. The study was approved by the University of British Columbia Children's and Women's Research Ethics Board and registered in advance with ClinicalTrials.gov (NCT03672565; https://clinicaltrials.gov/ct2/show/NCT03672565). Figure 1 provides an overview of participant flow through study procedures.

#### Recruitment Procedures

Study information was disseminated to local clinicians and community organizations, *via* online advertisements, and to suitable patients who completed an assessment through the Provincial OCD Program (POP), a tertiary level specialty clinic for OCD at BC Children's Hospital. Upon initial contact, study procedures and inclusion/exclusion criteria were discussed with families. Those interested and determined as likely eligible provided parental consent and youth assent to participate. Families referred by the POP provided consent for their clinical assessment data to be utilized for the present study to minimize study burden. External families completed a telephone screen and, if still eligible, progressed to an in-person diagnostic assessment.

#### Eligibility Criteria

Participants were youth between 7 and 19 years of age with a primary diagnosis of OCD who were seeking treatment and lived within an hour's drive of the study site. In order to be eligible, youth and at least one parent had to be willing to participate in treatment regardless of group assignment. Youth were required to have at least moderate symptom severity as indicated by a total score of 16 or greater on the Children's Yale-Brown Obsessive Compulsive Scale (CY-BOCS) (23). Participants were excluded if they were identified as having other mental health challenges that were a higher treatment priority than OCD or that posed a risk to participation in the study (e.g., extreme reactions to distress, self-harm). Youth were required to be on a stable medication regime (i.e., at least 10 weeks since initiation of a new serotonin reuptake inhibitor (SRI) and/or at least 4 weeks since initiation or dose adjustment of any existing psychotropic medication) and were restricted from receiving other interventions during active study treatment.

#### Treatment Phase I

Eligible families were randomized to treatment setting. In order to reduce potential bias, a computer-generated list that maintained a 1:1 condition assignment ratio over blocks of 4 or 6 participants was utilized. Following randomization, participants entered the first phase of study treatment. In the first phase, families received 3 × 3-h sessions. The first session comprised completion of a baseline assessment and an introduction to treatment while the following two-sessions were focused primarily on treatment delivery (see section Treatment Description for specifics). This initial dose was selected based on evidence that a portion of youth experience meaningful response after similarly brief interventions (10, 16). At the study outset, all three sessions were completed within a 7-day period (*n* = 12); however, to address emergent feasibility concerns in this pilot trial (e.g., difficulty staffing, higher burden on families), sessions were transitioned to occur weekly (*n* = 14). Following completion of Phase I, participants completed online surveys and were assessed by an independent evaluator (IE) who was blind to participants' group assignment. Participants achieving remission (i.e., CY-BOCS score < 11) (24) were transitioned to the follow-up phase of the study, while youth who had not yet achieved remission were offered the opportunity to enter Phase II.

#### Treatment Phase II

Families transitioned to Phase II were eligible to access up to four additional 3-h treatment sessions. Dosing in the second phase was selected in an effort to balance practical considerations (e.g., resource allocation, participant flow) with evidence suggesting a portion of youth require more substantial support to achieve treatment response (9). Each week, at least 72 h (3 days) prior to a potential session, families indicated their preference between: (A) completing another session; (B) ending treatment and transitioning to follow-up; or (C) delaying the decision by 1 week. Each family was provided with two opportunities to delay the decision, following which they were required to either utilize any remaining sessions or transition to follow-up. This provided families with scheduling flexibility (e.g., holidays, time to evaluate progress) while ensuring participant progression through the study. It also closely aligns with standard practice patterns in community-based treatment. Following either utilization of all four additional sessions or an earlier decision to end treatment, participants completed online surveys and a second assessment with the IE before moving to follow-up.

#### Follow-Up Phase

In the month following treatment completion, families were provided with up to three 30-min phone calls focused on reviewing, developing, and problem-solving independent ERP tasks, identifying next steps and long-term goals, supporting access to post-study services, and relapse prevention. This approach was selected based on simplicity, feasibility, and timing to follow-up. One- and six-months following treatment completion, participants completed online surveys and an additional assessment with the IE. Following completion of the one-month assessment, participants were free to access any other treatment resources, including medication changes.

### Measures

#### Demographic Information

Demographic information, as presented in **Table 2**, was provided by the primary caregiver organizing study participation on behalf of the youth. While a variety of response options were provided for demographic variables, **Table 2** presents relevant categories based on endorsed responses.

#### Eligibility Assessment

Participant OCD symptoms and severity were assessed using the CY-BOCS (23) while presence/absence of comorbid disorders was assessed *via* either: (A) the Anxiety Disorders Interview Schedule for DSM-IV Child Version (ADIS-C) for Parent Report (25) administered by MA-level clinicians under the supervision of a PhD-level psychologist (for externally referred participants); or (B) a comparable semi-structured interview completed by a PhD-level psychologist with expertise in OCD and related comorbidities (for POP assessed participants). For all participants, diagnoses were confirmed *via* group discussions involving PhD-level psychologists and child and adolescent psychiatrists.

#### Outcome Measures

See Table 1 for a detailed overview of all outcome measures included in the present study.

**Table 1:** Outcomes measures included in the present study.

**Domain**	**Measure name**	**Abbr**.	**Construct**	**Rater**	**Items**	**Scoring**	**Relevant citations**
OCD-related outcomes	Children's Yale-Brown Obsessive-Compulsive Scale—Severity Ratings	CY-BOCS	Youth's severity of symptoms caused by OCD	Clinician	10	0 (none)−4 (extreme)	(23, 26)
	Child Obsessive-Compulsive Impact Scale—Revised	COIS-R	Youth's level of impairment from OCD in home, school, and social functioning.	Parent^a^	33	0 (not at all)−3 (very much)	(27)
				Youth			
	OCD Family Functioning Scale—Part 1	OFF	Impacts of OCD on family routine, socio-occupational/school, and emotional functioning	Parent^a^	21	0 (never)−3 (daily)	(28, 29)
				Youth			
	Family Accommodation Scale—Self Report	FAS-SR	Family member engagement in OCD-related accommodations	Parent^a^	19	0 (none or not at all)−4 (everyday or extreme)	(30)
	Coercive Disruptive Behavior Scale for Pediatric OCD	CD-POC	Youth's distinctive coercive disruptive behaviors in the context of pediatric OCD	Parent	18	0 (never)−4 (almost all the time)	(31)
Secondary outcomes	Pediatric Quality of Life Enjoyment and Satisfaction Questionnaire	PQ-LES-Q	Youth's quality of life	Youth	15	1 (very poor)−5 (very good)	(32)
	Iowa Conners Rating Scale	IOWA	Inattentive, impulsive, and overactive (I-O) as well as oppositional and defiant (I-D) symptoms in youth	Parent	10	0 [not at all−3 (very much)]	(33)
	Revised Child Anxiety and Depression Scale	RCADS	Comorbid anxiety and depressive symptoms in youth	Parent^a^	47^b^	1 (never)−4 (always)	(34, 35)
				Youth			
	Child Avoidance Measure	CAM	Youth's avoidance of stimuli eliciting anxiety, fear or worry	Parent	8	0 (almost never)−3 (almost always)	(36)
				Youth			
Treatment perspectives	Treatment Perspective Form		Perspectives on treatment utility, quality and format	Parent^a^	10	0 (disagree)−100 (agree)	n/a
				Youth			

a *The measure was provided to two parents; however, given inconsistent completion among second parents, the average of available parent scores was utilized for outcomes*.

b *The six items from the obsessive-compulsive subscale were excluded from calculation of the total score*.

### Treatment Description

Study treatment was provided by masters-level clinicians under the supervision of the first author, a PhD-level psychologist with expertise in OCD-treatment.

#### Initial Session/Baseline Assessment

The first session focused on rapport building, baseline assessment of primary symptoms including re-completion of the CY-BOCS with the assigned study therapist, provision of psychoeducation, exploration of motivation and goal identification, treatment planning (hierarchy building), initial introduction to ERP, and homework planning. Youth and their parent(s) also completed baseline questionnaires online prior to the session.

#### Additional Sessions

Integrating current conceptualizations of evidence-based CBT for OCD (15, 37–39), subsequent sessions operated under the central principle that ERP is the key ingredient to effective treatment of OCD, while acknowledging that an individual must be willing to engage in the process for it to be effective (i.e., not coerced; not engaged in avoidance, distraction, or compulsions during ERPs). Consistent with this, treatment sessions focused primarily on ERP development, delivery, and homework planning, with flexibility to utilize other evidence-based cognitive-behavioral strategies to enhance engagement and address patient reluctance, avoidance, and non-compliance with homework (e.g., values identification, acceptance, distress tolerance).

Similarly, given extensive and varied impacts of OCD on family and the relevance of family variables to outcomes (e.g., accommodation, conflict) (28, 40–43), family members participated in homework review and planning at a minimum, with additional involvement (e.g., observation and participation in ERP) and direct support provided based on child developmental level and openness, as well as individual family needs. Common family supports included addressing accommodations, exploring and addressing relevant parent emotions and beliefs, behavior management skills (e.g., positive reinforcement, limit setting), and communication and relationship skills (e.g., validation, autonomy support).

Supplementary Table 1 provides an overview of average time spent on individual components per session as rated by the treating clinician following session completion. No significant differences were found between groups in regard to time spent on components.

### Analytic Plan

Statistical analyses were conducted using R software version 4.0.2. Following calculation of baseline descriptive statistics for the entire sample and each treatment group separately, treatment effects on repeatedly-measured outcome variables were evaluated using linear mixed-effects models. The intention-to-treat principle was followed such that all randomized participants were analyzed according to their treatment group allocation. The outcome of interest was modeled as a change from baseline at each follow-up time point. Treatment condition, time point, child age at baseline, and baseline value on the outcome variable were included as fixed effects. Additionally, the interaction between treatment condition and time point was included to evaluate differences between groups at each follow-up time point. Each model included a random intercept. These analyses use restricted maximum likelihood estimation, and all randomized participants contribute to estimation of treatment effects regardless of whether they complete follow-up assessments. In the results section, we present the estimated change from baseline within each group, the estimated difference between each group at each follow-up time point, and the 95% confidence intervals for these estimates.

Three binary outcomes, based on established definitions of response and remission (24), were calculated and reported descriptively using counts and percentages at each time point and for each group: (1) the number of youth who demonstrated > 35% reduction in CY-BOCS score from baseline at each timepoint; (2) the number of youth who demonstrated > 55% reduction in CY-BOCS score from baseline at each timepoint; and (3) the number of youth whose CY-BOCS score < 11 at the timepoint. Prior to calculating these values, missing CY-BOCS scores at each post-baseline follow-up were imputed using predictive mean matching, in which baseline age, treatment group, and prior CY-BOCS score was used to estimate the missing CY-BOCS total. Given that significance testing was already conducted on the continuous CY-BOCS measure, these outcomes are presented in a descriptive manner, with no additional statistical testing completed in regard to these findings.

Self- and parent-reported treatment perspectives were assessed only once (either at 1-month follow-up or during the booster call), and therefore, between-group differences were evaluated using analysis of covariance, with baseline age as a covariate and treatment group as the effect of interest.

## Results

### Sample Characteristics

Table 2 presents summary descriptive data for the entire randomized sample (*n* = 26) and separately for children randomly allocated to the Hospital setting group (“Hosp”; *n* = 14) and to the Home/Community setting group (“Home”; *n* = 12). The mean age at baseline was 14.1 years (SD = 2.5) and 52% of the sample identified as male. Generally consistent with local population demographics, the sample was composed of White and/or Asian families. No parents self-identified as having an OCD diagnosis.

**Table 2:** Baseline descriptive statistics for the full sample and within groups.

	**Overall (*****n*** **=** **26)**		**Hospital (*****n*** **=** **14)**		**Home (*****n*** **=** **12)**	
**Variable**	***n* (%) or mean (SD)**	**Missing, *n***	***n* (%) or mean (SD)**	**Missing, *n***	***n* (%) or mean (SD)**	**Missing, *n***
Child gender, male, *n* (%)	14 (56%)	1	6 (46%)	1	8 (67%)	0
Child age at screening	14.4 (2.7)	1	14.8 (2.3)	1	13.9 (3.1)	0
Age of first OC symptoms	10.0 (3.2)	1	9.7 (3.5)	1	10.4 (2.8)	0
Age at diagnosis	12.8 (2.8)	5	12.7 (2.8)	4	12.8 (2.9)	1
Age at worst OC symptoms	11.4 (3.4)	1	11.0 (3.9)	1	11.9 (3.0)	0
Ethnicity		1		1		0
East Asian	3 (12%)		1 (8%)		2 (17%)	
South Asian	3 (12%)		2 (16%)		1 (8%)	
West Asian	1 (4%)		1 (8%)		0 (0%)	
White (non-Hispanic/Latinx)	15 (60%)		7 (54%)		8 (67%)	
White (Hispanic/Latinx)	2 (8%)		2 (16%)		0 (0%)	
Mixed (East Asian/Caucasian)	1 (4%)		0 (0%)		1 (8%)	
Father's highest level of education, *n* (%)		1		1		0
High school or less	2 (8%)		2 (15%)		0 (0%)	
Community, technical, or trade degree	7 (28%)		3 (23%)		4 (33%)	
Undergraduate degree	10 (40%)		6 (46%)		4 (33%)	
Advanced degree	6 (24%)		2 (15%)		4 (33%)	
Mother's highest level of education, *n* (%)		1		1		0
High school or less	2 (8%)		1 (8%)		1 (8%)	
Community, technical, or trade degree	4 (16%)		1 (8%)		3 (25%)	
Undergraduate degree	11 (44%)		7 (54%)		4 (33%)	
Advanced degree	8 (32%)		4 (31%)		4 (33%)	
**Comorbidities, current**
Total combined, median (IQR)	0.5 (0, 2)	0	1.5 (0, 2)	0	0 (0, 2.25)	0
GAD, *n* (%)	10 (39%)	0	7 (50%)	0	3 (25%)	0
Social phobia, *n* (%)	3 (12%)	0	3 (21%)	0	0 (0%)	0
Separation anxiety, *n* (%)	1 (4%)	0	0 (0%)	0	1 (8%)	0
Specific phobia, *n* (%)	5 (19%)	0	1 (7%)	0	4 (33%)	0
Panic disorder, *n* (%)	1 (4%)	0	1 (7%)	0	0 (0%)	0
PTSD, *n* (%)	1 (4%)	0	1 (7%)	0	0 (0%)	0
Tics disorder, any, *n* (%)	3 (12%)	0	1 (7%)	0	2 (17%)	0
ADHD, *n* (%)	5 (19%)	0	3 (21%)	0	2 (17%)	0
Major depressive disorder, *n* (%)	1 (4%)	0	1 (7%)	0	0 (0%)	0
ASD, *n* (%)	1 (4%)	0	1 (7%)	0	0 (0%)	0
Prior psychosocial treatment for OCD	15 (60%)	1	9 (64%)	0	6 (55%)	1
SRIs, *n* (%)	9 (36%)	1	5 (36%)	0	4 (36%)	1

*Means (and standard deviations) are shown unless specified otherwise. Percentages are based on total of sample with available data*.

### Session Utilization

See Figure 1 for a detailed overview of session utilization and treatment decisions. Of the 26 youth who entered treatment, one youth in the hospital condition dropped out prior to completion of Phase I in order to resume treatment with their community provider while a participant in the home condition dropped out after utilizing two additional sessions in Phase II due to a desire to initiate medication given continued difficulty tolerating triggers, particularly outside of session (e.g., intense distress, aggressive behaviors). An additional two youth in the hospital condition were unable to complete the study due to interruptions associated with the onset of the COVID-19 pandemic and related restrictions. Of the remaining 22-youth who completed the study as intended, youth used a median 2.5 Phase II sessions [interquartile range (IQR): 1, 4], with a median of 2 in the hospital condition (IQR: 0.5, 4) and 3 in the home condition (IQR: 1, 4). Five youth (23%) utilized the minimum number of sessions while nine youth (41%) utilized all four additional sessions.

### Treatment Outcomes

**Figure 2:**
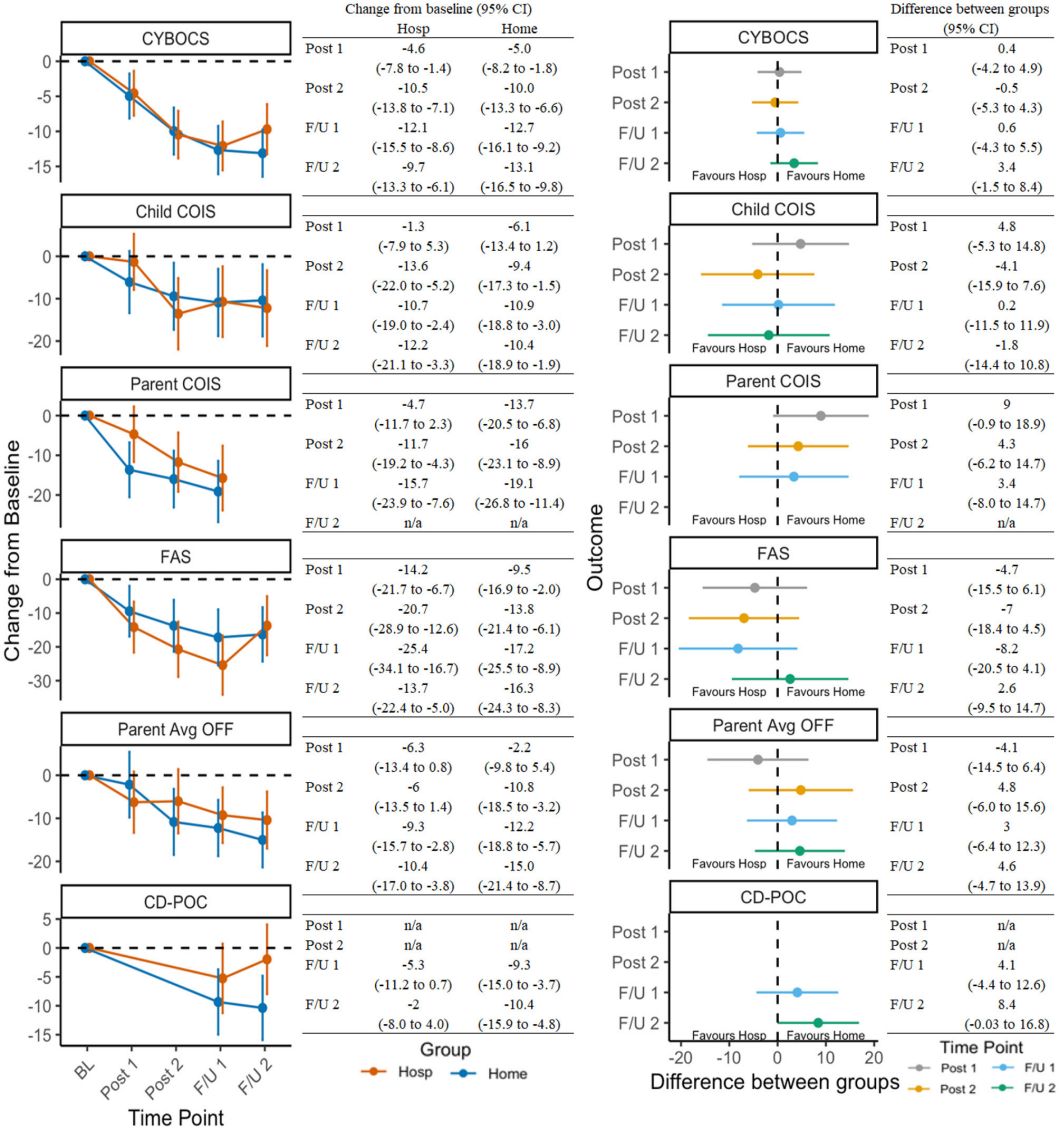
Change in OCD-related outcomes across timepoints and between groups. See Table 1 for a list of all measure abbreviations.

Supplementary Table 2 presents the observed mean scores and standard deviations for the 16 continuous outcomes at each time point for the Hospital and Home/Community groups. Figure 2 presents: (a) on the right half, the modeled change over time for the two treatment groups on OCD-related outcomes; and (b) on the left half, between-group difference at each of the follow-up time points for those same outcomes. As shown in the left half of Figure 2, for many of these outcomes there was a statistically significant change from baseline for both groups, indicated by mean estimates (dots) and confidence intervals (vertical lines) that do not cross the dashed horizontal line at zero. In particular, symptom severity, child impairment, family accommodation, and family functioning demonstrated significant improvements with relative consistency across setting assignment. Significant reductions in coercive/disruptive behaviors were also observed within the home, but not the hospital, condition (Figure 2, left half). In direct comparisons, no significant between-group differences were observed (Figure 2, right half).

**Figure 3:**
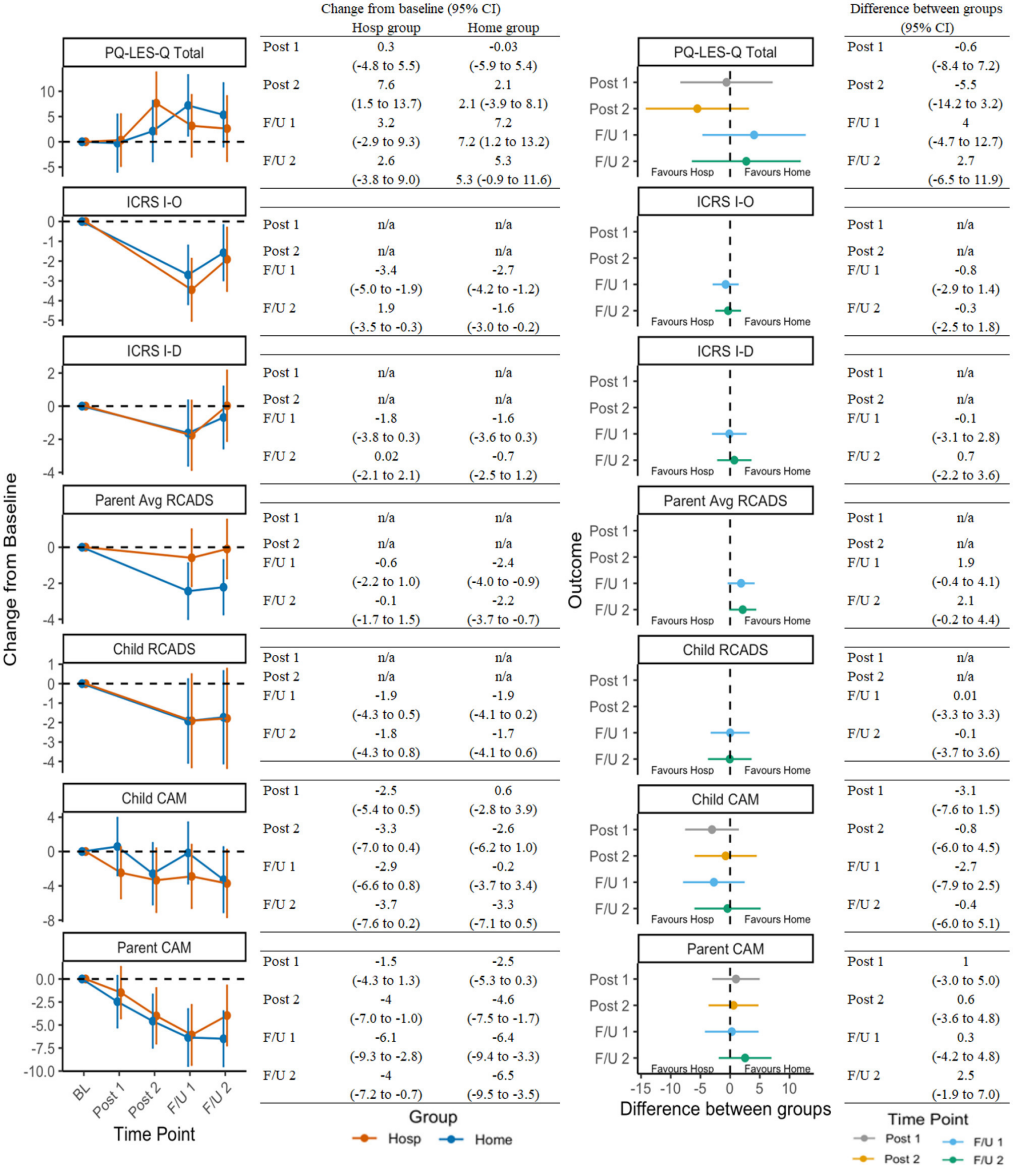
Change in secondary outcomes across timepoints and between groups. See Table 1 for a list of all measure abbreviations.

Figure 3 presents secondary outcomes in the same manner as OCD-outcome representation in Figure 2. Improvements in secondary domains were less robust or consistent, although results still suggested treatment was associated with reductions in avoidance, improvements in quality of life, and reductions in comorbid symptoms (e.g., anxiety/depression, attention deficit-hyperactivity disorder; ADHD).

Table 3 shows the proportion of youth within each group meeting standardized definitions of response and remission based on the CY-BOCS absolute scores and score changes. Differences between groups suggest more favorable outcomes within the home condition, particularly at 6-month follow-up.

**Table 3:** Levels of response and remission within groups at each time point.

	**Post 1**		**Post 2**		**Follow-up 1**		**Follow-up 2**	
**Outcome, *n* (%)**	**Hosp**	**Home**	**Hosp**	**Home**	**Hosp**	**Home**	**Hosp**	**Home**
Response (35% reduction in CY-BOCS)	2 (14%)	1 (8%)	7 (50%)	5 (42%)	11 (79%)	9 (75%)	8 (57%)	11 (92%)
Remission (55% reduction in CY-BOCS)	0 (0%)	1 (8%)	1 (7%)	2 (17%)	3 (21%)	6 (50%)	1 (7%)	6 (50%)
Remission (CYBOCS ≤ 11)	0 (0%)	1 (8%)	2 (14%)	3 (25%)	3 (21%)	6 (50%)	1 (7%)	6 (50%)

### Treatment Satisfaction

Table 4 presents the between-group differences in treatment perspectives, as reported by ratings from the child and the average of two parents. As noted above, these data were collected only at one follow-up session. The program was rated highly overall by both youth and parents. Youth in the home condition rated treatment significantly more favorably in regard to ease of completion and pleasantness. In contrast, items related to recommending the program to others and supporting the program being made a permanent service were rated higher by parents in the hospital group. Youth and parents in the home condition were significantly more likely to report that they believed they benefited more in their assigned treatment setting than they would have in the other condition.

**Table 4:** Youth and parent perspectives regarding treatment with comparisons between groups.

	**Child perspectives**			**Parent avg. perspectives**		
**Aspect of treatment^**a**^**	**Hosp *M* (SE)**	**Home *M* (SE)**	**Diff (95% CI)**	**Hosp *M* (SE)**	**Home *M* (SE)**	**Diff (95% CI)**
Easy to understand	66.9 (8.9)	89.7 (8.3)	−22.8 (−49.7 to 4.1)	90.8 (8.0)	76.7 (7.0)	14.1 (−9.6 to 37.7)
Easy to complete	39.4 (7.3)	62.6 (6.8)	−23.2 (−45.5 to −0.9)^*^	79.0 (5.8)	73.7 (5.1)	5.3 (−11.9 to 22.5)
Pleasant	32.8 (9.0)	69.8 (8.4)	−37.1 (−64.4 to −9.8)^*^	71.1 (8.1)	73.2 (7.1)	−2.2 (−26.0 to 21.7)
Helpful	73.1 (11.1)	77.6 (10.3)	−4.5 (−38.2 to 29.2)	94.2 (5.2)	83.2 (4.9)	11.0 (−5.1 to 27.1)
Convenient	57.9 (11.0)	83.1 (10.3)	−25.2 (−58.7 to 8.3)	81.1 (6.3)	78.1 (5.5)	3.0 (−15.6 to 21.5)
Relevant to symptoms	69.0 (9.4)	82.0 (8.8)	−12.9 (−41.6 to 15.7)	94.5 (4.1)	84.4 (3.6)	10.0 (−2.1 to 22.2)
Worth time/effort	78.8 (11.4)	83.8 (10.6)	−5.0 (−39.7 to 29.6)	89.1 (7.7)	83.7 (6.8)	5.4 (−17.4 to 28.2)
Recommend to others	86.9 (4.6)	96.2 (4.3)	−9.4 (−23.4 to 4.6)	100.0 (1.86)	90.5 (1.6)	9.1 (3.6 to 14.6)^*^
Should be permanent service	84.0 (5.6)	94.1 (5.2)	−10.0 (−26.9 to 4.6)	100.0 (2.4)	88.4 (2.1)	11.2 (4.1 to 18.3)^*^
Condition was important to success	52.6 (8.0)	22.6 (7.4)	30.0 (5.7 to 54.3)^*^	44.2 (5.8)	19.7 (5.4)	24.5 (6.8 to 42.2)^*^

* *Significantly different between groups*.

a *Items were rated from 0 (totally disagree)−100 (totally agree) with the exception of the last item which was rated from 0 (would have benefited less in other condition)−100 (would have benefited more in other condition)*.

## Discussion

This randomized pilot study investigated the benefits of an intensive flexible-length CBT program while comparing outcomes across home and hospital setting delivery. Consistent with prior research, the intensive CBT program was efficacious, with large reductions observed across OCD-specific domains as well as modest benefits in more global domains (comorbid symptoms, quality of life). Observed differences in treatment session utilization levels across participants suggest that flexibility in treatment dosing is desirable and useful in optimizing levels of care based on individual need. Treatment was rated highly by participants overall. The present study provides further evidence that intensive CBT is a feasible, desirable, and efficacious form of treatment for pediatric OCD.

Both groups demonstrated comparable reductions in symptom severity and no between group differences were statistically significant. Youth in the hospital condition utilized a median of one fewer sessions than those in the home condition, which could indicate a faster rate of change, but may also have been influenced by youth's dislike for the hospital setting or reduced utility of remaining in treatment given more limited opportunities for ERP completion within the hospital setting. Although lower median session utilization within the hospital condition may play a role, close inspection of the data suggest some potential benefits associated with the home condition. In particular, reductions in coercive/disruptive-behaviors were significant within the home condition but not the hospital condition; the home condition demonstrated slightly larger improvements in youth- and family-functioning when rated by parents; and rates of response and remission favored the home condition, particularly by the time of 6-month follow-up. Youth and families within this condition may have benefited from additional opportunities to tackle symptoms and impairment within their natural environment and develop alternative systems of management/response, potentially enhancing generalizability and maintenance of learning. In contrast, families among the hospital condition initially demonstrated greater reductions in family accommodation, which may reflect how, when opportunities for specific-ERPs are limited by setting (e.g., touching the bed), clinicians instead can support families around reducing accommodations related to those symptoms and achieve positive outcomes. This trend was no longer evident at 6-month follow-up.

Despite limited between-group differences, youth in the home condition rated treatment more favorably (significant for easier and more pleasant), and both youth and their parents in the home condition were more likely to report that they believed they benefited more from being in their assigned condition than they would have in the alternative setting. In comparison, parents in the hospital condition appeared to rate treatment more positively (significant for recommending to others and belief that program should be a permanent service). Our clinical observations suggested that home conduct of sessions added particular value for certain participants (e.g., for whom primary triggers or impairments were focused within the home) while being less relevant to others (e.g., for whom primary triggers were internal or exhibited impairment in non-home settings). Without consideration of inter-individual contextual influences on OCD, the potential to identify benefits associated with home-treatment may be notably diluted. Overall, home-based work may increase participant buy-in and willingness, allow for more naturalistic experiences for patient/family learning, and have particular utility for context-dependent symptoms. Given this, a blended model that incorporates standard clinic-based service provision with occasional in-home/community ERP sessions, especially when relevant, may be optimal for minimizing costs/therapist burden while still capitalizing on potential benefits of home-based sessions.

The present study contributes to the growing body of evidence supporting a transition away from fixed-length individual treatment models toward patient-driven treatment and supports intensive CBT as a suitable format to provide tailored care. In particular, the extended session length facilitated in-home treatment provision and increased ERP engagement and practice, allowing for substantial within-session progress and rapid improvements within a short period of time. However, clinical observations and informal participant feedback indicate that greater flexibility (even beyond that offered in the current pilot study) is warranted. First, for families with limited scheduling flexibility, lower levels of impairment/immediate need, and/or higher levels of ambivalence, the 3-h format may be a barrier to accessing and/or continuing with treatment. As a result, the traditional 1-h session length may be optimal for many families. Second, we observed emergent challenges around treatment decisions when families had largely improved but still desired supports around specific symptoms that could not be effectively targeted in session (e.g., bedtime ritual). This challenge could likely be addressed by tapering down from 3- to 1-h sessions as symptoms improve. Third, while the total therapeutic dose in the present study was limited to a maximum of 22.5-h (7 × 3 h + 3 × 0.5 h), many families requested, and would likely have benefited from continued treatment or higher levels of care, as has been demonstrated previously (9, 17).

The following are limitations of the present study. First, eligibility assessments differed slightly depending on recruitment source which may have impacted determination of eligibility and identification of comorbid conditions. Second, given the use of a flexible treatment protocol, lack of a control condition, and limits to participant choices (e.g., no 1-h session options; defined maximum amount of treatment offered), the present study did not aim to assess superiority relative to standard weekly sessions, or to establish an optimal approach to dosing. Third, as a pilot trial, the study focused primarily on establishing the overall feasibility and efficacy of the treatment program (regardless of delivery method) and lacked power to detect smaller between group differences. Given some indications toward the potential benefit of home-delivered treatment (particularly when clinically-relevant), further study of this domain is warranted. Fourth, responding to feasibility concerns, Phase I session frequency was changed mid-study, potentially introducing an additional confound; however, the impacts of this confound should be equivalent across groups. Fifth, facilities used for treatment delivery within the hospital condition were limited and generally less comfortable or inviting than may be typical of community-based offices (e.g., medically oriented, small, undecorated). This may have contributed to less favorable perceptions within this condition, such as in regards to “pleasantness.” Finally, the extent to which findings may generalize to other groups may be limited by the characteristics of the sample (e.g., ethnicity, gender identity, caregiver type).

In summary, the results of the study support the conclusions that: (1) intensive CBT is an efficacious treatment for pediatric OCD that produces improvements in a wide variety of domains and is acceptable to patients and their families; (2) adjusting the amount of treatment provided based on patient need/preference is feasible and allows for flexible allocation of resources; and (3) although treatment setting was not found to have a major impact on outcomes, treating patients within their home environment may offer some additional benefits in generalizability and maintenance of gains as well as youth satisfaction. Continued efforts to develop and evaluate individualized approaches to the treatment of pediatric OCD are warranted.

## Data Availability Statement

The datasets presented in this article are not readily available because sharing of data is dependent on participant consent that data may be shared (either exclusively within Canada or internationally). Requests to access the datasets should be directed to S. Evelyn Stewart, estewart@bcchr.ca.

## Ethics Statement

The studies involving human participants were reviewed and approved by University of British Columbia Children's and Women's Research Ethics Board. Written informed consent to participate in this study was provided by the participants' legal guardian/next of kin.

## Author Contributions

RS, LF, and SES contributed to conception and design of the study. ZN managed study recruitment, implementation, and data collection. RS, DF-Y, SQ, JF, XD, and DH completed initial assessments and delivered study treatment. CO, LB, and JN completed blinded study assessments. RS, JN, and SES provided supervision and study oversight. JB performed the statistical analysis. RS, ZN, and JB, wrote sections of the manuscript. All authors contributed to manuscript revision, read, and approved the submitted version.

## Conflict of Interest

The authors declare that the research was conducted in the absence of any commercial or financial relationships that could be construed as a potential conflict of interest.
