# Supplementary material for: Effects of Treatment Setting on Outcomes of Flexibly-Dosed Intensive Cognitive Behavioral Therapy for Pediatric OCD: A Randomized Controlled Pilot Trial
Source: Front Psychiatry. 2021 May 17;12:669494. doi: 10.3389/fpsyt.2021.669494 (PMC8165233; doi:10.3389/fpsyt.2021.669494)
Supplement: Supplementary file 1 [file Table_1.docx]

Supplemental Table 1. Average number of minutes per 180-minute session spent on treatment components.

|  | **Overall (N = 26)** | **Home (n = 12)** | **Hosp (n = 14)** | **Group Difference^1^** |
| --- | --- | --- | --- | --- |
| **Core Components** | *M (SD)* | *M (SD)* | *M (SD)* | *p-value* |
| Update & Homework Review | 18 (4) | 19 (4) | 17 (4) | 0.2 |
| ERP Discussion and Planning | 14 (7) | 15 (7) | 13 (8) | 0.5 |
| Imaginal ERP | 24 (16) | 21 (11) | 27 (19) | 0.4 |
| In Vivo ERP | 94 (26) | 92 (25) | 95 (28) | 0.7 |
| Homework Planning | 18 (5) | 19 (5) | 18 (5) | 0.6 |
| **Additional Components**^2^ |  |  |  |  |
| General Troubleshooting | 6 (7) | 7 (7) | 6 (7) | 0.6 |
| Motivation Building | 10 (9) | 10 (11) | 11 (6) | 0.8 |
| Review of Psychoeducation | 11 (7) | 11 (9) | 11 (6) | > 0.9 |
| Parent-Focused Coaching | 7 (7) | 9 (8) | 6 (5) | 0.4 |
| Crisis Management | 1 (2) | 1 (2) | 1 (2) | 0.5 |
| Addressing Non-OCD Domains | 2 (3) | 2 (3) | 3 (3) | 0.5 |

*^1^Welch Two Sample t-test.* ^2^*Time may have overlapped with provision of core components.*
