# Supplementary material for: Effects of Treatment Setting on Outcomes of Flexibly-Dosed Intensive Cognitive Behavioral Therapy for Pediatric OCD: A Randomized Controlled Pilot Trial
Source: Front Psychiatry. 2021 May 17;12:669494. doi: 10.3389/fpsyt.2021.669494 (PMC8165233; doi:10.3389/fpsyt.2021.669494)
Supplement: Supplementary file 2 [file Table_2.docx]

Supplemental Table 2. Descriptive statistics for time-varying continuous measures.

| Timepoint  Condition | | | | **Baseline** | | **Post-1** | | **Post-2** | | **1-Month** | | **6-Month** | |
| --- | --- | --- | --- | --- | --- | --- | --- | --- | --- | --- | --- | --- | --- |
|  |  |  |  | **Hosp** | **Home** | **Hosp** | **Home** | **Hosp** | **Home** | **Hosp** | **Home** | **Hosp** | **Home** |
| **Domain** | **Outcome** | **Measure** | **Rater** | Mean (SD)  [% of group with data] | | | | | | | | | |
| OCD-Related Outcomes | Severity | CYBOCS | Clinician | 25.21 (5.18)  [100%] | 24.92 (3.12)  [100%] | 20.69 (5.25)  [93%] | 20.25 (4.63)  [100%] | 15.27 (4.00)  [79%] | 15.70 (6.24)  [83%] | 13.20 (3.97)  [71%] | 11.78 (6.44)  [75%] | 15.88 (8.15)  [57%] | 11.90 (4.58)  [92%] |
|  | Child Impairment | COIS-R | Child | 26.00 (22.41)  [100%] | 24.36 (13.62)  [92%] | 22.54 (15.37)  [93%] | 18.30 (11.88)  [83%] | 11.38 (6.91)  [57%] | 13.78 (14.19)  [75%] | 12.29 (7.20)  [50%] | 12.75 (12.24)  [67%] | 12.00 (7.46)  [43%] | 14.29 (11.16)  [67%] |
|  |  |  | Parent 1 | 30.64 (20.50)  [100%] | 32.33 (11.85)  [100%] | 25.31 (17.52)  [93%] | 17.75 (11.76)  [100%] | 20.91 (15.58)  [79%] | 16.27 (9.63)  [92%] | 18.11 (16.71)  [64%] | 11.67 (13.80)  [75%] | - | - |
|  | Family Impairment | OFF | Parent Avg | 32.61 (12.43)  [100%] | 29.79 (12.66)  [100%] | 23.57 (10.72)  [50%] | 20.80 (11.71)  [42%] | 20.83 (10.87)  [43%] | 16.20 (14.81)  [42%] | 19.95 (12.91)  [71%] | 16.06 (12.09)  [75%] | 20.81 (19.10)  [57%] | 14.00 (12.76)  [92%] |
|  | Family Accommodation | FAS | Parent 1 | 32.93 (16.37)  [100%] | 23.83 (14.40)  [100%] | 16.00 (12.69)  [93%] | 16.00 (19.79)  [100%] | 10.55 (11.53)  [79%] | 12.64 (14.24)  [92%] | 6.11 (7.36)  [64%] | 5.44 (6.00)  [75%] | 15.50 (18.90)  [57%] | 6.90 (8.45)  [92%] |
|  | Coercive and Disruptive Beh | CD-POC | Parent 1 | 18.36 (11.38)  [100%] | 14.00 (13.16)  [100%] | - | - | - | - | 7.89 (10.68)  [64%] | 4.11 (5.09)  [75%] | 13.12 (16.08)  [57%] | 3.60 (4.81)  [92%] |
| Secondary Outcomes | Quality of Life | PQ-LES-Q | Child | 48.29 (9.15)  [100%] | 52.09 (9.66)  [92%] | 48.85 (8.32)  [93%] | 51.00 (10.25)  [83%] | 57.12 (5.96)  [57%] | 53.89 (10.30)  [75%] | 52.00 (8.47)  [50%] | 59.62 (11.36)  [67%] | 50.50 (10.82)  [43%] | 58.00 (9.11)  [67%] |
|  | ADHD Symptoms | ICRS I-O | Parent 1 | 6.43 (3.63)  [100%] | 5.67 (2.87)  [100%] | - | - | - | - | 3.00 (2.87)  [64%] | 2.89 (3.41)  [75%] | 5.38 (3.81)  [57%] | 4.60 (4.09)  [92%] |
|  | Oppositional Symptoms | ICRS I-D | Parent 1 | 5.43 (3.55)  [100%] | 4.08 (2.84)  [100%] | - | - | - | - | 3.11 (2.80)  [64%] | 2.33 (2.74)  [75%] | 5.25 (4.98)  [57%] | 3.70 (3.68)  [92%] |
|  | Child Anxiety | RCADS | Parent Avg | 8.19 (3.27)  [100%] | 6.70 (2.81)  [100%] | - | - | - | - | 5.91 (1.66)  [71%] | 4.90 (1.67)  [75%] | 7.05 (3.88)  [57%] | 5.11 (1.75)  [92%] |
|  |  |  | Child | 9.61 (5.63)  [100%] | 9.04 (3.52)  [92%] | - | - | - | - | 6.43 (2.51)  [50%] | 6.77 (4.20)  [67%] | 6.73 (3.11)  [43%] | 7.49 (3.17)  [67%] |
|  | Child Avoidance | CAM | Child | 13.21 (5.49)  [100%] | 14.45 (4.55)  [92%] | 10.23 (3.75)  [93%] | 13.90 (6.42)  [83%] | 8.62 (4.84)  [57%] | 9.00 (6.61)  [75%] | 8.14 (3.85)  [50%] | 12.50 (5.71)  [67%] | 7.83 (4.07)  [43%] | 11.29 (7.13)  [67%] |
|  |  |  | Parent 1 | 12.00 (7.40)  [100%] | 14.00 (7.30)  [100%] | 10.85 (6.48)  [93%] | 10.83 (6.83)  [100%] | 8.91 (5.19)  [79%] | 9.18 (6.91)  [92%] | 6.67 (4.77)  [64%] | 5.33 (4.74)  [75%] | 7.88 (6.47)  [57%] | 6.00 (5.81)  [92%] |

Notes: CY-BOCS: Children’s Yale-Brown Obsessive-Compulsive Scale – Severity Ratings; COIS-R: Child Obsessive-Compulsive Impact Scale – Revised; OFF: OCD Family Functioning Scale – Part 1; FAS: Family Accommodation Scale – Self Report; CD-POC: Coercive Disruptive Behaviour Scale for Pediatric OCD; PQ-LES-Q: Pediatric Quality of Life Enjoyment and Satisfaction Questionnaire; ICRS I-O: Iowa Conners Rating Scale – Inattentive, Impulsive & Overactive Subscale; ICRS I-D: Iowa Conners Rating Scale – Oppositional & Defiant Subscale; RCADS: Revised Child Anxiety and Depression Scale; CAM: Child Avoidance Measure
